# Cilostazol Ameliorates Peripheral Neuropathic Pain in Streptozotocin-Induced Type I Diabetic Rats

**DOI:** 10.3389/fphar.2021.771271

**Published:** 2022-01-18

**Authors:** Kuang-I. Cheng, Hung-Chen Wang, Kuang-Yi Tseng, Yi-Hsuan Wang, Chung-Yu Chang, Yi-Jing Chen, Chung-Sheng Lai, Dar-Ren Chen, Lin-Li Chang

**Affiliations:** ^1^ Department of Anesthesiology, Kaohsiung Medical University Hospital, Kaohsiung, Taiwan; ^2^ Graduate Institute of Clinical Medicine, College of Medicine, Kaohsiung Medical University, Kaohsiung, Taiwan; ^3^ Department of Neurosurgery, Chang Gung Memorial Hospital-Kaohsiung Medical Center, Chang Gung University College of Medicine, Kaohsiung, Taiwan; ^4^ Department of Microbiology and Immunology, College of Medicine, Kaohsiung Medical University, Kaohsiung, Taiwan; ^5^ Department of Surgery, Division of Plastic and Reconstructive Surgery, Kaohsiung Medical University Hospital, Kaohsiung, Taiwan; ^6^ Endoscopic and Oncoplastic Breast Surgery Center, Changhua Christian Hospital, Changhua, Taiwan; ^7^ Department of Surgery, Division of General Surgery, Changhua Christian Hospital, Changhua, Taiwan; ^8^ Graduate Institute of Medicine, College of Medicine, Kaohsiung Medical University, Kaohsiung, Taiwan; ^9^ Department of Medical Research, Kaohsiung Medical University Hospital, Kaohsiung, Taiwan

**Keywords:** cilostazol, neuropathic pain, diabetes, voltage-gated sodium channel, glial cells

## Abstract

**Background:** Cilostazol is an antiplatelet agent with vasodilating, endothelial function restoration, and anti-inflammatory effects. This study aims to investigate the efficacy of oral cilostazol for preventing the development of diabetic peripheral neuropathy (DPN).

**Materials and Methods:** Ninety adult male Sprague-Dawley rats were divided into five groups: 1) naïve (control); 2) diabetic (DM); 3) DM receiving 10 mg/kg cilostazol (cilo-10); 4) DM receiving 30 mg/kg cilostazol (cilo-30); and 5) DM receiving 100 mg/kg cilostazol (cilo-100). Hindpaw responses to thermal and mechanical stimuli were measured. Activation of microglia and astrocytes in the spinal dorsal horn (SDH) and expression of NaVs in the dorsal root ganglia (DRG) were examined with Western blots and immunofluorescence.

**Results:** DM rats displayed decreased withdrawal thresholds to mechanical stimuli (mechanical allodynia) and blunted responses to thermal stimuli. In addition, the expression of microglia increased, but astrocytes were reduced in the SDH. Upregulation of Nav −1.1, 1.2, −1.3, −1.6, and −1.7 and downregulation of Nav-1.8 were observed in the DRG. The DM rats receiving cilostazol all returned DM-induced decrease in withdrawal threshold to mechanical stimuli and attenuated neuropathic pain. Additionally, all cilostazol treatments suppressed the level of activated microglial cells and ameliorated the DM-induced decline in astrocyte expression levels in the SDH. However, only the rats treated with cilo-100 demonstrated significant improvements to the aberrant NaV expression in the DRG.

**Conclusion:** Oral cilostazol can blunt the responses of mechanical allodynia and has the potential to treat diabetic neuropathy by attenuating NaV and glial cell dysregulation.

## Introduction

Diabetic peripheral neuropathy (DPN) is a debilitating complication affecting up to 60% of patients with types I and II diabetes mellitus (DM) (Standl et al., 2019). It is characterized by various peripheral nerve abnormalities and dysfunctions due to chronic hyperglycemia that contributes to hyper- or hyposensitizations of the peripheral organs (Freeman et al., 2016). These include apparent endoneurial edema of the sciatic and sural nerves and significant reductions in motor nerve conduction velocity observed in patients with DM and in experimental models (Jakobsen 1978; Giannini and Dyck, 1994). The latter is largely due to irregular nerve phenotypes, including total endoneurial lipid concentration reduction, myelinated fiber loss, and increased frequencies of denatured Schwann cells and regenerating fibers (Brown et al., 1979; Giannini and Dyck 1994; Cermenati et al., 2012). Voltage-gated sodium channels (NaVs) are critical for the initiation and propagation of action potentials and are essential for the transmission of noxious stimuli in the nociceptive neurons. Peripheral nerve damage, inflammation, and metabolic diseases alter the expression and function of these NaVs, leading to increases in neuronal excitability and pain. The NaV subunits −1.3, −1.7, and 1.8 are well known for their distinctively altered expression and repriming profiles in injured neurons and STZ-induced diabetic rats (Cheng et al., 2014; Bennett et al., 2019). The normally slow-activating and repriming NaV-1.7 exhibited significantly higher expression and similar nerve injury-induced inflammatory pain and hypersensitivity in cases of sciatic nerve axotomy, (Rush et al., 2007; Dib-Hajj et al., 2017; Bennett et al., 2019). Distinct elevations in NaV-1.3 were also observed in both the small and large DRG neurons of rat models with spinal nerve contusion, sciatic nerve axotomy, and type I DM (Bennett et al., 2019). Modulation of the peripheral NaVs expression and activity is a promising avenue for neuropathic pain management.

Cilostazol is a selective phosphodiesterase (PDE)-3 inhibitor that has demonstrated antiplatelet, antithrombotic, vasodilatory, and anti-proliferative effects on smooth muscle cells *in vivo* (Chapman and Goa 2003; Asal and Wojciak 2017). It was originally recommended for treating intermittent claudication, but has also emerged as an option for treating other vascular dysfunctions (Donnelly 2002; Thompson et al., 2002; Chapman and Goa 2003). Recent studies demonstrated that oral cilostazol improved the overall vascular health of hindlimb ischemia in mice by increasing the levels of endothelial progenitor cells in the circulation, as well as those of granulocyte colony-stimulating factor and vascular endothelial growth factor in the ischemic muscles (Biscetti et al., 2013). Although cilostazol does not appear to affect the blood glucose levels in people with DM, it is effective in modulating the levels of albuminuria and hyperglycaemia-induced metabolic abnormalities (Matsumoto et al., 2008; Tang et al., 2013). Additionally, cilostazol effectively manages cellular inflammation and oxidative stress, provides renoprotection, and reduces the coronary heart disease risk in DM (Agrawal et al., 2007; Wang et al., 2008; Lee et al., 2020). In an *in vitro* study, cilostazol demonstrated the ability to protect human endothelial cells against lipopolysaccharide-induced apoptosis via the ERK-1/2 and p38 MAPK-dependent pathways (Lim et al., 2008). In 2007, Iwama et al. (2007) reported that oral cilostazol effectively reduced the levels of neuronal injury and subsequent retinal injury in rats with cerebral ischemia by inhibiting ischemia-induced interactions between the leukocytes and endothelial cells (Iwama et al., 2007). Koyanagi et al. reported that cilostazol could suppress Schwann cell dedifferentiation by promoting cyclic AMP (cAMP) signaling via PDE inhibition, suggesting its potential in ameliorating chemotherapy-induced peripheral neuropathy (Koyanagi et al., 2021).

DPN is a major chronic complication of DM. Its pathogenesis mainly involves chronic glucose toxicity and nerve ischemia. Apart from its positive effects on vascular and endothelial abnormalities, information on the benefits of cilostazol on peripheral nerve function remains limited. The action of cilostazol on the peripheral nerve includes increased nerve blood flow and restoration of nerve Na⁺/K⁺-ATPase activity, leading to improved sciatic-tibial nerve conduction velocity and amelioration of DPN (Kihara et al., 1995; Naka 1995). In addition, the neuroprotective effects of cilostazol include the ability to promote axonal regeneration in the sciatic nerves of DM rats (Yamamoto et al., 1998). This study aimed to identify effective oral cilostazol dosages for preventing the development of DPN via various pathways including regulating the peripheral sodium channels, glial cell activation in the spinal cord, and alleviation of diabetic neuropathic pain.

## Materials and Methods

### Cilostazol Preparations and Administrations

Cilostazol (tablet, OTSUKA PHARMACEUTICAL CO., LTD., Japan) was crushed and resuspended in ddH2O as either 20 mg/ml or 50 mg/ml stock aliquots and administered daily via oral gavage at either 10, 30, or 100 mg/kg by referring to publications (Naka 1995; Rosales et al., 2011) from the second week of successful DM induction for up to 6 weeks until sacrifice.

### Animals and Diabetes Induction

Ninety adult male Sprague-Dawley rats weighing 250–300 g were used in this study. All rats were housed in plastic cages with soft bedding and maintained under a 12-h light-dark cycle regime (light cycle, 7 am–7 pm; dark cycle, 7 pm–7 am), with access to food and water *ad libitum*. All experimental procedures were approved by the Kaohsiung Institutional Animal Care and Use Committee (Approval No. 106008). Diabetes was induced by administration of 60 mg/kg streptozotocin (STZ, Sigma, St. Louis, MO, United States) via the femoral vein (Lee et al., 2011; Cheng et al., 2014). The rats were divided into five groups: 1) naïve (control), surgery to expose the right femoral vein, and intravenous injection of normal saline); 2) diabetic (DM), surgery to expose the right femoral vein and intravenous injection of 60 mg/kg STZ; 3) DM plus 10 mg/kg cilostazol (cilo-10), right femoral vein injection of 60 mg/kg STZ and oral cilostazol 10 mg/kg daily for 6 weeks; 4) DM plus 30 mg/kg cilostazol (cilo-30), right femoral vein injection of 60 mg/kg STZ and oral cilostazol 30 mg/kg daily for 6 weeks; and 5) DM plus 100 mg/kg cilostazol (cilo-100), right femoral vein injection of 60 mg/kg STZ, and oral cilostazol 100 mg/kg daily for 6 weeks. Successful induction of diabetes was confirmed via elevation of random blood glucose levels to over 500 mg/dl using an Accu-Chek^®^ Performa blood glucose assay kit. The rats were sacrificed at day 56, the L5 DRG and spinal cords were removed.

### Behavioral Responses to Thermal and Mechanical Stimuli

The rats were subjected to electrical von Frey and heat plantar tests to assess the animals’ sensitivity to mechanical and thermal stimuli, respectively. Prior to each test, the rats were acclimated to the respective environments for testing for up to 30 min. Hypersensitivity to mechanical and thermal stimuli in the hindpaw was assessed as described in our previous study (Cheng et al., 2014). The testing facility for mechanical allodynia assessment consisted of a metal mesh floor covered by a transparent plastic dome (8 × 8 × 18 cm). For the measurements of hindpaw withdrawal thresholds against mechanical stimuli, a Dynamic Plantar Aesthesiometer (UgoBasile, Italy) with an incremental increase of 2.5 g/s and a maximum cut-off threshold of 50 g was used. The withdrawal threshold of each paw was calculated as the average of four to six tests. To measure the latency of hindpaw withdrawal from a heat stimulus, each hindpaw was set on a glass plate heated at 193 mW/cm^2^ by a directed infrared light beam through a pinhole of 2 × 5 cm, emitted from a moveable light box (UgoBasile Model 7370, Italy). The thermal stimulus was terminated either by withdrawal of the paw from the glass plate or by automation at a 20-s cut-off time. The withdrawal threshold of each paw was calculated as the average of four to five continuous tests, with a minimum of 5 minutes’ rest between each test.

### Protein Extractions and Western Blots

For protein extraction, frozen L5 DRG samples were homogenized in a commercially available RIPA buffer (Invitrogen cat. 89901) containing a complete protease inhibitor mixture (Roche Diagnostics GmbH, Mannheim, Germany). For Western blots of the NaVs, 50 µg of total protein from each sample was loaded onto 8% sodium dodecyl sulfate-polyacrylamide gels (SDS-PAGE) and transferred to polyvinylidene fluoride membranes (PVDF, Millipore, Bedford, MA). The filters were blocked with 5% milk in phosphate-buffered saline (PBS) with 0.1% Tween 20 for 1 h at room temperature and incubated for 24 h at 4 C with rabbit anti-rat Navs primary antibodies (Alomone Labs, Jerusalem, Israel) included Nav-1.1 (ASC-001), Nav-1.2 (ASC-002), Nav-1.3 (ASC-004), Nav-1.6 (ASC-009), Nav-1.7 (ASC-008), Nav-1.8 (ASC-016), and mouse anti-rat *ß*-actin (MilliporeSigma, MAB1501). This was followed by a reaction with horseradish peroxidase-conjugated mouse anti-rabbit (Santa Cruz Biotechnology, sc-2357) or rabbit anti-mouse secondary antibodies (Santa Cruz Biotechnology, sc-358914) to detect the expression of NaVs in the DRG. The intensity of each band was visualized using ECL Western blotting detection reagents (Amersham Biosciences, Tokyo, Japan) and captured using the UVP ChemiDoc-It^®^ 810 Imager system (P/N 97–0645-05, 100–115 V–60 Hz, United Kingdom). NaVs expression were normalized using *β*-actin. Quantification of NaVs expression levels was normalized against the NaVs levels in control rats.

### Histological Samples Preparations and Immunofluorescence

The dissected L5 DRG and spinal cord tissues were fixed in 4% (w/v) paraformaldehyde and then saturated in 10–30% (w/v) sucrose in 0.02 mol/L PBS (pH 7.4). Once the samples were sufficiently dehydrated in 30% sucrose solutions, they were embedded into an optimal cutting temperature compound (FSC; FSC22 Clear, Surgipath, Leica) in preparation for subsequent cryosectioning procedures. Sections of the DRG (12-µm) and spinal cord (30-µm) were cut using a cryostat and mounted onto glass slides for immunostaining of the NaVs and glial cells. The expression of NaV −1.1, −1.2, −1.3, −1.6, −1.7 and −1.8 protein was detected using rabbit anti-NaVs primary antibodies (the same antibodies used in Western blotting) overnight at 4 C. The slides were then incubated with Cy3-conjugated goat anti-rabbit (Chemicon, Temecula, CA, United States) secondary antibodies for 1 h at 37 C. To visualize the activated microglia and astrocytes in the spinal cord, polyclonal goat anti-Iba1 primary antibody (Abcam, ab5076) and polyclonal goat anti-GFAP primary antibody (Abcam, ab53554) were used. After overnight incubation at 4°C, Cy3 donkey anti-goat IgG secondary antibody (Jackson ImmunoResearch, 765–165-147) was added for 2 h at 37 C. The stained sections were examined and images were captured using an Olympus FluoView 1000 confocal laser scanning microscope (Olympus, Tokyo, Japan). Quantification measurements of immunofluorescence staining in the spinal cord were performed as described in our previous report (Cheng et al., 2014).

### Statistical Analysis

Western blots were determined by one-way ANOVA analysis followed by the least significant difference test for multiple post hoc analyses. Behavioral responses, microglia, and astrocyte activation were assessed using the Mann–Whitney *U* test. SPSS 20.0 (SPSS Inc., Chicago, IL) was used for all statistical analyses. Statistical significance was set at *p < 0.05, **p < 0.01, and ***p < 0.001.

## Results

### Cilostazol Alleviated Mechanical Allodynia in Diabetic Rats

**FIGURE 1:**
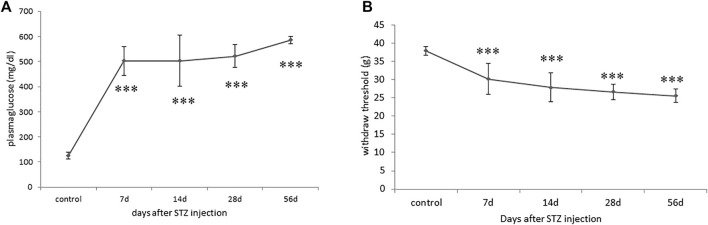
Progression of blood glucose levels and mechanical allodynia phenotype in streptozotocin (STZ)-induced diabetic (DM) rats. **(A)** Hyperglycaemia indicated by significant increases in blood glucose levels of DM rats throughout the 2-month experimental periods. **(B)** A significant reduction in levels of hindpaw withdrawal thresholds against mechanical stimuli in DM rats indicated the development of mechanical allodynia. Results are expressed as mean SEM for a minimum of 10 rats. Statistical significances between the baseline control and DM groups were calculated with the Mann–Whitney *U* test. ***p < 0.001.

A single intravenous STZ injection induced persistent hyperglycemia in rats within 7 days of operation, with their random blood glucose levels maintained at approximately 400–600 mg/dl, indicating successful DM induction (Figure 1A). Mechanical allodynia was observed on postoperative day 7 and persisted for 2 months among the successfully induced DM rats, as indicated by the significant reductions in hindpaws withdrawal thresholds against mechanical stimuli (Figure 1B). However, STZ-induced diabetic rats demonstrated blunt responses to thermal stimuli (data not shown).

**FIGURE 2:**
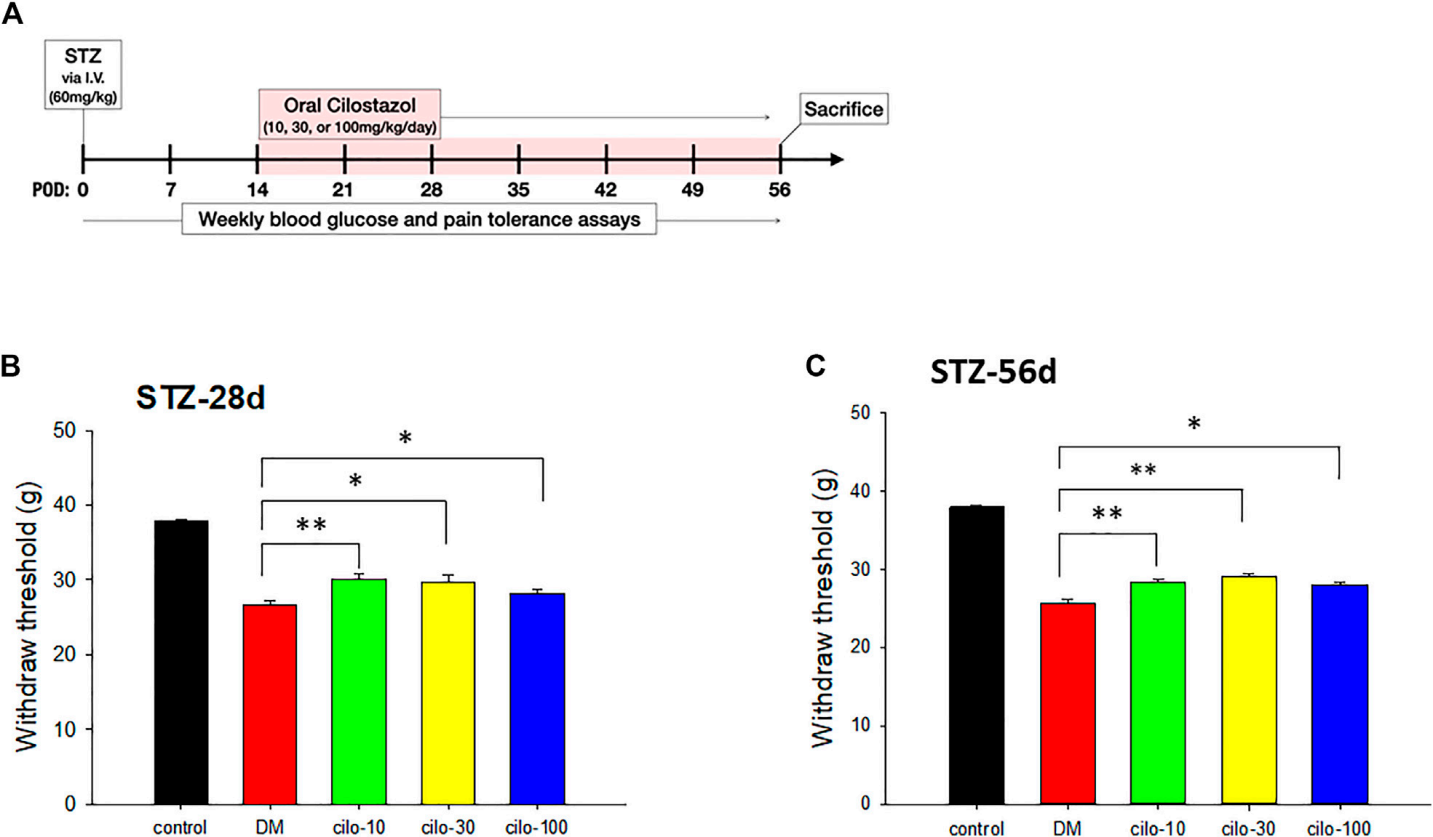
Analgesic effect of cilostazol in streptozotocin (STZ)-induced diabetic (DM) rat model. **(A)** Experiment timelines outlining relative time points of intravenous (I.V.) STZ injection, commencement of daily cilostazol oral gavage, and sacrifice. Significant increases in tolerance against mechanical stimuli in **(B)** 28-days (two weeks cilostazol) and **(C)** 56-days (six weeks cilostazol) DM rats indicated by higher levels of hindpaw withdrawal thresholds. POW, postoperative week. Results are expressed as mean SEM for a minimum of five rats for each group. Statistical significances between the DM control and cilostazol DM treatment groups were calculated with the Mann–Whitney *U* test. *p < 0.05, **p < 0.001.

2 weeks after STZ injection, the rats were administered 10, 30, or 100 mg/kg cilostazol daily via oral gavage for 6 weeks to investigate the efficacy of cilostazol in ameliorating neuropathic pain (Figure 2A). All three dosages of cilostazol significantly restored the hindpaws’ mechanical stimulus responses as compared with 1-month DM rats (Figure 2B), as well as 2-month DM rats (Figure 2C). The results indicated that even a low dose (10 mg/kg) of cilostazol could alleviate STZ-induced mechanical allodynia.

### High-Dose Cilostazol is Necessary to Alleviate Streptozotocin-Induced Dysregulated Sodium Channels in the Dorsal Root Ganglia

**FIGURE 3:**
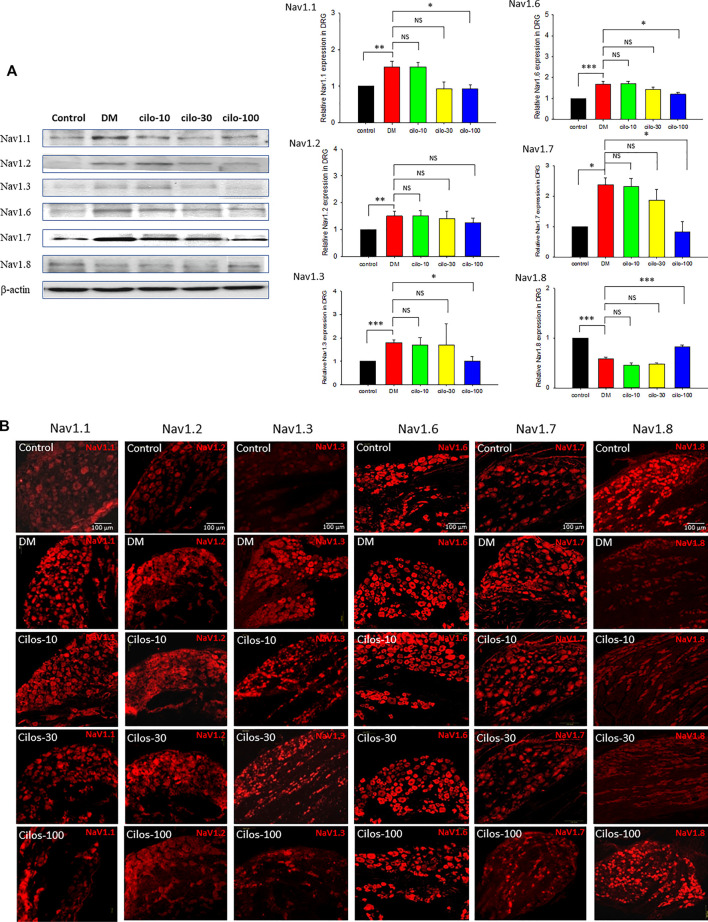
Dose-dependent recovery of voltage-gated sodium (NaV) channel expression in dorsal root ganglia (DRG) of cilostazol-treated diabetic (DM) rats. Expressions of NaV-1.1, -1.2, -1.3, -1.6, -1.7, and -1.8 in DRG of naïve control, DM, and oral cilostazol (10, 30, and 100 mg/kg) treated DM rats were examined with **(A)** Western blots and **(B)** immunofluorescence assays. The western and immunofluorescence data revealed significant upregulation of NaV-1.1–1.7 expressions, and significant downregulation of NaV-1.8 in the DRGs of DM rats. Daily oral cilostazol treatments of 100 mg/kg, but not the lower dosages, for 6 weeks significantly reversed the DM-induced NaV dysregulation in the DRGs. Results are expressed as mean SEM for a minimum of five rats for each group. Statistical significances between the DM control and cilostazol DM treatment groups were calculated by one-way ANOVA analysis followed by the least significant difference test for multiple post hoc analyses. **p < 0.01, *p < 0.05, ***p < 0.001.

As in our previous study (Cheng et al., 2014), persistent hyperglycemia caused abnormal NaVs expression in the L5 DRG. Upregulation of NaV −1.1, −1.2, −1.3, −1.6, −1.7, and downregulation of NaV-1.8 were observed in the DRG. However, among the three cilostazol-treated groups, only the high-dose (100 mg/kg) cilostazol administration significantly alleviated dysregulated NaVs expression except in the case of NaV-1.2 (Figures 3A,B) in DRG.

### Cilostazol Alleviated Streptozotocin-Induced Dysregulated Glial Cells in Spinal Cord

**FIGURE 4:**
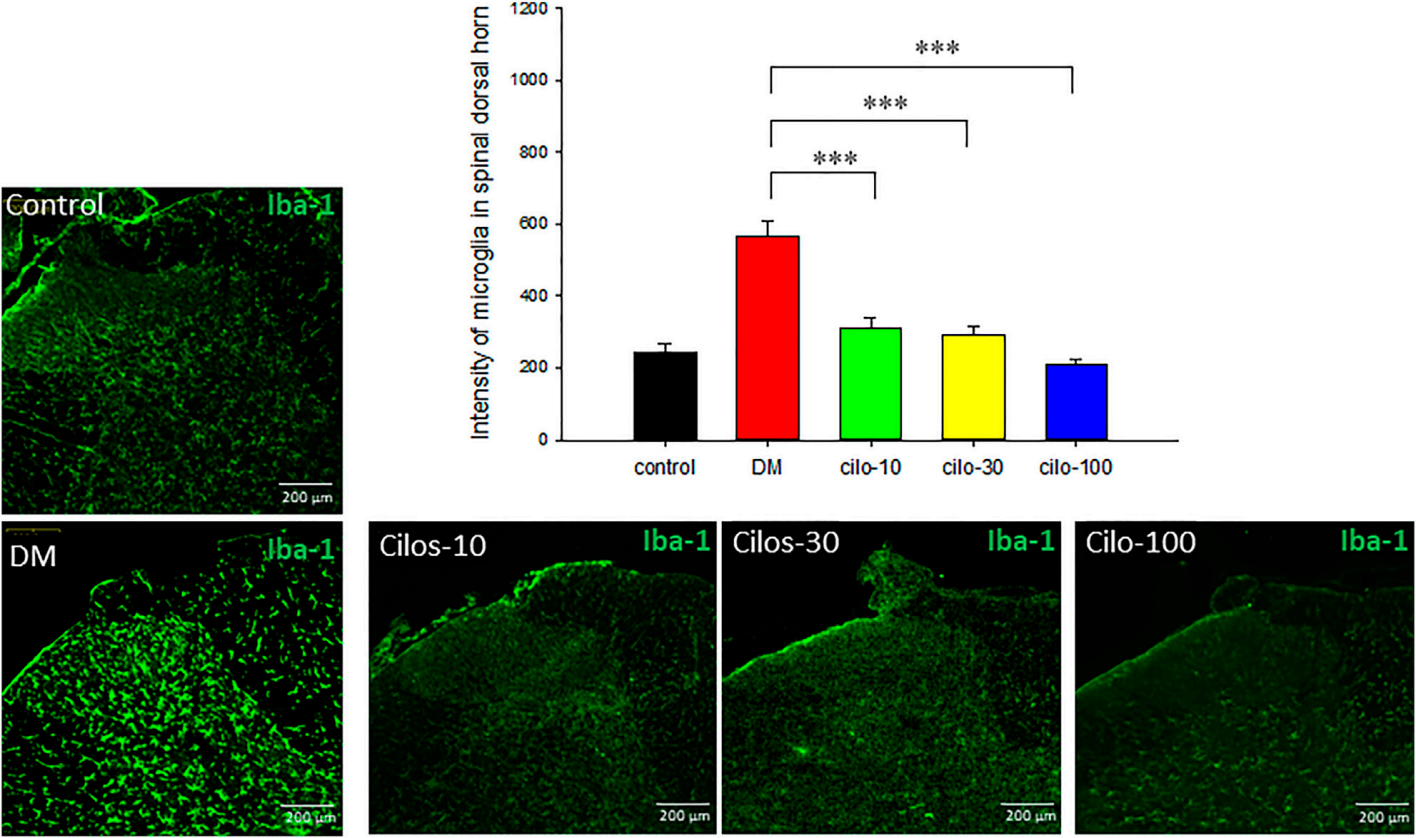
Oral cilostazol significantly reduced the levels of activated microglia expression in the spinal dorsal horn (SDH) of diabetic (DM) rats. Immunofluorescence assays revealed increased expression of activated microglia (Iba-1; green) in the SDH of 2-month streptozotocin-induced DM rats, and reduced expressions in the cilostazol-treated DM rats. Samples from DM rats treated with all three oral cilostazol dosages demonstrated significantly reduced levels of activated microglia (intensity of green fluorescence) in the SDH. Results are expressed as mean SEM for a minimum of five rats for each group. Statistical significances between the DM control and cilostazol DM treatment groups were calculated with the Mann–Whitney *U* test, ***; *p* < 0.001.

**FIGURE 5:**
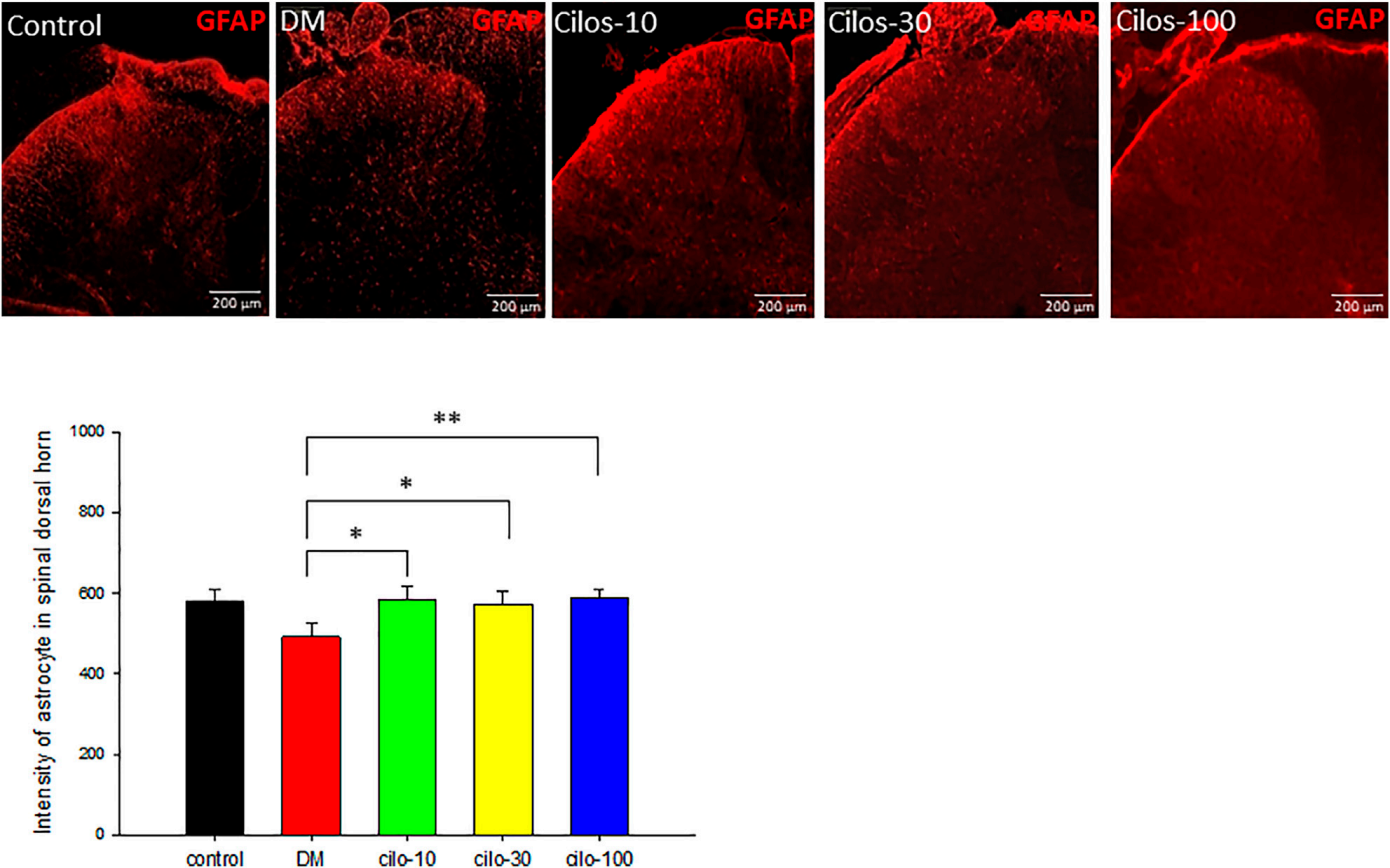
Cilostazol ameliorated levels of activated astrocytes in the spinal dorsal horn (SDH) of streptozotocin (STZ)-induced diabetic (DM) rats. Expressions of astrocytes in the SDH were detected using GFAP (red) via immunofluorescence. All cilostazol-treated rats demonstrated significantly higher levels of astrocytes expressions in the SDH compared to the DM samples. Results are expressed as the mean SEM for a minimum of five rats for each group. Statistical significances between the DM control and cilostazol DM treatment groups were calculated via the Mann–Whitney *U* test. *p < 0.05, **p < 0.01, ***p < 0.001.

Diabetic rats displayed significantly increased activation of microglia in SDH compared to the control group (Figure 4), whereas astrocytes exhibited reduced expression in the SDH (Figure 5). After cilostazol administration, glial cell dysactivation was significantly ameliorated regardless of whether the dose was low (10 mg/kg) or high (100 mg/kg) dose (Figures 4, 5). Low-dose cilostazol is sufficient to attenuate the immunoreactivity of activated microglia and restore the DM-induced decrease in the immunoreactivity of astrocytes.

## Discussion

The present study revealed that consistent daily cilostazol administration for 6 weeks influenced glial cell expression in SDH, and NaVs in DRGs, to approach approximately normal levels and provide an analgesic effect. Importantly, low-dose (10 mg) cilostazol was sufficient to suppress the aberrant excitability of SDH glial cells and alleviate neuropathic pain, although high-dose (100 mg) cilostazol was necessary to restore the expression of NaVs in the DRG. Our results indicate that cilostazol has vasodilatory, anti-inflammatory, and renoprotective actions, and can be used as an alternative agent in treating diabetic neuropathy with neuropathic pain.

The variable etiology of peripheral neuropathic pain in diabetic neuropathy is characterized by chronic hyperglycemia. Recent knowledge of the mechanisms causing DPN and the generation of neuropathic pain in DM remains incomplete. Sodium channels are critical determinants of sensory neuronal excitability, associated neuropathic pain signals, and peripheral neuropathy (Cardoso and Lewis 2018). Patients with genetic variants of sodium channels are at risk of neuropathic pain during the development of diabetes (Lauria et al., 2014). Our current and previous studies demonstrated significant increases in the levels of NaV-1.1, −1.2, −1.3, −1.6, and −1.7, and reduction in NaV-1.8 protein expression in DRG neurons in diabetic rats (Cheng et al., 2014). Similar findings have demonstrated that dysregulated sodium channels are associated with DRG hypersensitivity and DPN (Faber et al., 2012a; Faber et al., 2012b; Yang et al., 2016). Particularly, variants in the genes encoding for the NaV-1.7, −1.8, and −1.9 sodium channel subunits have been discovered in patients with small-fiber neuropathy and can lead to the development of pain (Lauria et al., 2014; Sopacua et al., 2019).

Glial cells, most notably astrocytes and microglia, which cooperate to promote and preserve neuronal health, play important roles in regulating the activity of neuronal networks in the brain. The activation of microglia secondary to neuroinflammatory processes contributes to the development and pain signaling through the activation of p38 MAPK or by expressing P2X4 receptors in microglia (Tsuda et al., 2003; Cheng et al., 2014). In our previous and present studies, the expression of microglia increased, and a positive correlation between mechanical allodynia, NaV-1.3, and microglial activation was observed in STZ-induced diabetic rats (Cheng et al., 2014). Furthermore, microglial activation is mediated by the phosphorylation of p-38 mitogen-activated protein kinase (Cheng et al., 2014).

Astrocytes are vulnerable to hypoxia under acidic conditions in diabetes. A previous study reported that hyperglycemic ischemia caused astrocyte activation in the early stage but astrocyte death in the late stage with enhanced free radical production (Muranyi et al., 2006). Another report stated that STZ leads to the activation of microglia and astrocytes in the DRG and spinal cord (Barragán-Iglesias et al., 2018; Tawfik et al., 2018). However, Zhang et al. addressed the decreased astrocyte expression in the spinal cord of diabetic rats (Cheng et al., 2014; Zhang et al., 2018). GFAP activation was reduced in the first 2 months and then returned to the level found in control rats at the third and 6 months in STZ-induced diabetic rats (Cheng et al., 2014). An interesting study by Liao reported that a db/db type 2 diabetes mouse model that displayed obvious mechanical allodynia was associated with the activation of spinal astrocytes but not microglia by the “Astrocyte-IL-1β-NMDAR-Neuron” pathway (Liao et al., 2011). In brief, increased activation of microglia in the spinal cord of diabetic rats has been consistently reported; however, the activation status of astrocytes in the spinal cord in STZ-induced diabetes was diverse. Further studies are needed to clarify the role of astrocytes in DPN.

Apart from facilitating good glycemic control, tricyclic antidepressants, serotonin-noradrenaline reuptake inhibitors, and anticonvulsants are recommended as first-line drugs for DPN to reduce pain; however, these treatments remain inadequate (Khdour 2020). Voltage-gated sodium channels are another therapeutic target for the treatment of painful diabetic neuropathy (Knezevic et al., 2020). Sodium channel blockers have been investigated for years as a potential treatment for chronic pain (Zuliani et al., 2010; Urru et al., 2020). Drugs from natural products (Chopra et al., 2010; Tiwari and Chopra 2011) or traditional Chinese Medicine (Bai et al., 2019) with therapeutic potential on diabetic complications were also reported. Cilostazol at 30 mg/kg/day improves Na⁺/K⁺-ATPase activity, while cilostazol at 10 mg/kg/day increases intracellular cAMP levels in the peripheral nerves of diabetic rats (Naka 1995). These studies suggest that cilostazol may have potential in the treatment of diabetic neuropathy. The efficacy of cilostazol in the treatment of DPN has not been proven in humans; however, participants in a human clinical trial with DM with critical limb ischemia administered cilostazol for more than 3 months achieved significantly decreased amputation and mortality rates and had significantly better outcomes (Lee et al., 2020). Cilostazol 100 mg/d was effective in improving walking speed in patients with DM with neuropathy; however, no significant deterioration or improvement in motor and sensory nerve conduction parameters was observed (Rosales et al., 2011). In our present diabetic neuropathy animal study, cilostazol 100 mg/kg/day demonstrated significant improvements in aberrant NaV expression in the DRG. Based on our results, we suggest that cilostazol may be an alternative to restore sodium channels for treating neuropathic pain in diabetes-induced sodium channel dysfunction. The cellular mechanisms of cilostazol in microglia are poorly understood. According to published results, cilostazol suppresses the NF-kappa B, ERK, and JNK signaling pathways to inhibit pro-inflammatory cytokines such as TNF-alpha production in activated microglia (Yoshikawa et al., 1999; Jung et al., 2010). Our results indicated that cilostazol suppressed the levels of activated microglial cells and ameliorated the decreased astrocyte expression levels in the SDH. Cilostazol may act as an anti-inflammatory agent by downregulating diabetes-induced microglial activation and suppressing mechanical allodynia. Although improvements in DM-induced allodynia were observed at the lowest cilostazol dosage, our study demonstrated that consistent, high-dose oral cilostazol administration could not only ameliorate the symptoms of mechanical allodynia in STZ-induced DM rats, but could also reduce levels of changes in the key NaV proteins in the DRG. These results indicated that the glial cells are more sensitive to the effect of cilostazol than are neurons; in other words, cilostazol exerts a cell-based dosage disparity effect in the nervous system (Table 1).

**TABLE 1:** Summary of the differential responses of NaV proteins in dorsal root ganglia neurons and microlglia cells in spinal dorsal horns toward cilostazol treatments in streptozotocin-induced type I diabetic rats.

	T1 DM	T1 DM	T1 DM
		low-dose cliostazol	high-dose cliostazol
(DRG)			
NaV-1.1	↑	↑	−
NaV-1.2	↑	↑	↑
NaV-1.3	↑	↑	−
NaV-1.6	↑	↑	−
Nav-1.7	↑	↑	−
NaV-1.8	↓	↓	−
(SDH)			
lba1^+^ gilla cells	↑	−	−
GFAP^+^ astrocytes	↓	−	−

Upward arrows indicate higher values, downward arrows indicate lower values, and hyphens indicate similar values compared to normal controls.

In conclusion, we confirmed that treatment with cilostazol may attenuate diabetic neuropathic pain by inhibiting glial cell dysregulation and NaVs malfunction in the SDH and DRG, respectively. This pilot study provides the foundation for an animal study to clarify alternative targets of cilostazol for pain modulation in DNP.

## Data Availability

The original contributions presented in the study are included in the article, further inquiries can be directed to the corresponding author.
